# The Encapsulation of Febuxostat into Emulsomes Strongly Enhances the Cytotoxic Potential of the Drug on HCT 116 Colon Cancer Cells

**DOI:** 10.3390/pharmaceutics12100956

**Published:** 2020-10-11

**Authors:** Usama A. Fahmy, Hibah M. Aldawsari, Shaimaa M. Badr-Eldin, Osama A. A. Ahmed, Nabil A. Alhakamy, Helal H. Alsulimani, Filippo Caraci, Giuseppe Caruso

**Affiliations:** 1 Department of Pharmaceutics, Faculty of Pharmacy, King Abdulaziz University, Jeddah 21589, Saudi Arabia; uahmedkauedu.sa@kau.edu.sa (U.A.F.); haldosari@kau.edu.sa (H.M.A.); smbali@kau.edu.sa (S.M.B.-E.); oaahmed@kau.edu.sa (O.A.A.A.); nalhakamy@kau.edu.sa (N.A.A.); dr.helal@live.com (H.H.A.); 2 Department of Pharmaceutics and Industrial Pharmacy, Faculty of Pharmacy, Cairo University, Cairo 12613, Egypt; 3 Advanced Drug Delivery Research Group, Faculty of Pharmacy, King Abdulaziz University, Jeddah 21589, Saudi Arabia; 4 Center of Excellence for Drug Research and Pharmaceutical Industries, King Abdulaziz University, Jeddah 21589, Saudi Arabia; 5 Oasi Research Institute—IRCCS, Via Conte Ruggero, 73, 94018 Troina (EN), Italy; carafil@hotmail.com; 6 Department of Drug Sciences, University of Catania, 95125 Catania, Italy

**Keywords:** febuxostat, emulsomes, drug release, apoptosis, necrosis, cytotoxicity, colorectal cancer

## Abstract

Febuxostat (FBX) is a drug able to inhibit xanthine oxidase and reduce uric acid production commonly used for the treatment of hyperuricemia in subjects suffering from gout. Several studies have also been directed at its use as anti-cancer drug during the last years, opening a window for its off-label use. In the present study, an optimized formulation in terms of vesicle size and drug release, obtained by encapsulation of FBX into the emulsomes (FBX-EMLs), was evaluated for its cytotoxic potential in human colorectal carcinoma (HCT 116) cells. The optimized FBX-EMLs formula had an improved half maximal inhibitory concentration (IC50), about 4-fold lower, compared to the free drug. The cell cycle analysis showed a significant inhibition of the HCT 116 cells proliferation following FBX-EMLs treatment compared to all the other conditions, with a higher number of cells accumulating on G2/M and pre-G1 phases, paralleled by a significant reduction of cells in G0/G1 and S phases. The optimized formula was also able to significantly increase the percentage of cell population in both early and late stages of apoptosis, characterized by a higher intracellular caspase-3 concentration, as well as percentage of necrotic cells. Lastly, the FBX ability to decrease the mitochondrial membrane potential was enhanced when the drug was delivered into the EMLs. In conclusion, the new formulation of FBX into EMLs improved all the parameters related to the anti-proliferative activity and the toxic potential of the drug towards colorectal cancer cells.

## 1. Introduction

Cancer is a major health issue, as it involves several molecular mechanisms that cause uncontrolled proliferation due to abnormal cell signaling [1]. There are many risk factors for cancer development including abnormalities in genetics, epigenetics, family history, race, sex, obesity, low physiological activity, nutrition, radiation, and stress [2,3]. Based on the information available on the Global Cancer Observatory (GCO) (https://gco.iarc.fr/), an interactive web-based platform presenting global cancer statistics to inform cancer control and research, colorectal cancer occupies the second position for number of deaths (∼880 thousand; 9.2%), the third position for estimated number of new cases (∼1.85 million; 10.2%), and the second position with regard to the expected number of prevalent cases (5 years) (∼4.8 million; 10.9%) worldwide (Data source: GLOBOCAN 2018). By year 2040, the mortality rate as well as the incidence of this type of cancer is expected to rise significantly [4]. Several risk factors such as obesity, sedentary lifestyle, red meat consumption, androgen deprivation therapy, smoking, and alcohol intake are considered to contribute significantly to colorectal cancer development [5]. This type of cancer is often diagnosed at an advanced stage when the dissemination of tumor cells have already occurred, with a five-year survival prognosis strictly dependent on the stage of the disease (90% survival for patients with stage I vs. 10% survival for patients with stage IV) [6]. During the last decade, the standard strategy to treat colorectal cancer has been surgery and chemotherapy; however, the prognosis of this type of cancer has never been satisfying, especially with regard to patients undergoing metastatic lesions [7]. Efforts to improve current chemotherapy by adding more cytotoxic agents failed in enhancing patient survival and, in some cases, increased the side effects [8]. Based on the above, we investigated a new possible strategy to treat colorectal cancer by formulating molecules with known pharmacological use in other diseases (drug repositioning), then minimizing the required preclinical toxicological studies necessary before drug approval [9].

Febuxostat (FBX) was developed as a xanthine oxidase inhibitor to treat gout by reducing uric acid production. Unlike hypouricemic drugs, FBX does not have a purine structure [10,11]. As an anti-gout drug, FBX exists in different pharmacological formulations including emulsions, solid dispersion, and tablets [12,13,14]. Since FBX reduces uric acid production in the body, it has been used as a prophylaxis for prevention of tumor lysis syndrome in patients with malignant tumors receiving chemotherapy [15]. Recently, FBX showed cytotoxic effects in a tumor cell line (A549) inducing apoptotic events mediated by caspase 3 [16]. However, the specific mechanisms of cytotoxicity are still unknown. FBX is a weak acid drug with a pKa of 3.3 that under stomach acidic environment exists in its unionized form [17]. However, when it passes to small intestine, the alkaline environment increases the ionized form of FBX, which minimizes cell permeability and systemic bioavailability. Similarly, under neutral pH of blood, it will exist in its ionized form, which limits the drug availability to cells [17]. Therefore, FBX must be formulated to improve its cellular penetration and increase its cytotoxicity.

Emulsomes (EMLs) are vesicular lipoidal systems composed of solid fat inner core encircled by phospholipid multilayers [18,19,20]. The drug can be loaded in phospholipid layers and the inner core. The main feature of these nanocarriers is that the inner fat core is in a liquid crystalline or solid phase rather than remaining in a fluid oil phase [21]. This characteristic distinguishes EMLs from emulsions, also enabling for the encapsulation of a higher amount of drug. Due to the lipophilic nature of EMLs, cell permeation increases and, consequently, there is an improvement in the solubility, bioavailability, and efficacy of insoluble drugs [22,23]. Furthermore, EMLs are more stable compared with other well-known vesicular carriers such as liposomes, showing low toxicity profile, prolonged drug release, and improved drug efficacy [24,25]. These characteristics of EMLs make the base for the investigation of this nanocarriers to formulate FBX, with the aim to improve the cytotoxicity of this drug on HCT 116 colorectal cancer cells, a well-established in vitro human cell line model used in drug screenings and drug discovery programs in the field of cancer to predict clinical response to anti-cancer drugs [26,27,28,29].

As previously mentioned, we want to investigate new possible strategies to treat colorectal cancer (and make it the basis for other cancer types) by formulating molecules with known pharmacological use in other diseases. By doing so, we believe to achieve a double effect corresponding to the shortening of the necessary preclinical toxicological studies that characterize drug development/approval and significantly decrease or, in the best scenario, reduce the risk to develop side effects and safety problems. So with this in mind, the focus of our work was to investigate the ability of EMLs to improve the cytotoxic and apoptotic efficacy of FBX in human colorectal carcinoma (HCT 116) cells. A Box-Behnken test design was used to find the optimized formula with minimum vesicle size. The optimized FBX-EMLs were then subjected to in vitro drug release. Lastly, the FBX-EMLs were examined in HCT 116 cells for the determination of important parameters related to cytotoxicity including half maximal inhibitory concentration (IC50) values, cell cycle phases, the percentage of apoptotic and necrotic cell populations and caspase-3 concentration, and mitochondrial membrane potential (MMP) variation.

## 2. Materials and Methods

### 2.1. Materials and Reagents

FBX was a generous gift from Spimaco Addwaeih (Al-Qassim—Saudi Arabia). Phospholipon^®^ 90 H (hydrogenated soybean lecithin, containing at least 90% phosphatidylcholine (PC)) was a kind gift from Lipoid GmbH (Frigenstr, Ludwigshafen, Germany). All the remaining chemicals were of analytical grade and purchased from Sigma-Aldrich Corporate (St. Louis, MO, USA) or Thermo Fisher Scientific Inc. (Pittsburgh, PA, USA) unless specified otherwise.

### 2.2. Experimental Design of FBX-EMLs

Box-Behnken experimental design was utilized for FBX-EMLs formulation using Design-Expert software (Version 12; Stat-Ease Inc., Minneapolis, MN, USA). Three independent variables were investigated including two formulation variables, FBX concentration (% *w*/*w*, X_1_) and PC concentration (% *w*/*w*, X_2_), and one process variable, ultrasonication time (min, X_3_). The effect of the studied variables on the response, particle size (nm, Y), was studied. The coded levels of each factor designated as (−1, 0, +1) and their corresponding actual values are depicted in Table 1.

A total of 17 experimental runs, including 5 center points, were generated by the software. The levels of variables for each experimental run and its measured particle size are demonstrated in Table 2.

Model fit statistics was applied to select the best fitting model for the measured response based on the predicted and adjusted *R*^2^. The response was then statistically analyzed using analysis of variance (ANOVA) at *p* < 0.05. To explore the interaction between the studied variables, three dimensional surface plots were generated.

### 2.3. Preparation of FBX-EMLs

To prepare FBX-EMLs, specified amounts of FBX, PC, cholesterol (4% *w*/*v*), and tripalmitin (2% *w*/*v*) were dissolved in chloroform/methanol mixture (2:1, *v*/*v*) [20]. The organic solution was subjected to rotary evaporator under reduced pressure at 40 °C. The formed film layer in the round bottom flask was kept in a vacuum oven for 24 h to ensure complete removal of organic solvents from lipid film. The dried film was hydrated with 10 mL of phosphate buffer saline (PBS) (pH 5.5) and then ultra-sonicated (Sonics & Materials Inc., Newtown, CT, USA). FBX concentration, lipid concentration, and ultrasonication time were specified for each run as indicated by the Box-Behnken experimental design. The prepared FBX EMLs were kept at 4 °C until further investigation.

### 2.4. Measurement of Vesicle Size

The prepared FBX-EMLs were examined for vesicle size determination using a dynamic light scattering technique that was applied using a particle size analyzer (Zetasizer Nano ZSP, Malvern Panalytical Ltd., Malvern, UK). The prepared formulation (100 µL) was diluted in distilled water. The average size of five measurements were recorded.

### 2.5. Optimization of FBX-EMLs

Numerical method following desirability approach were utilized for the optimization process of the prepared FBX-EML formulations. The optimization process aimed at minimizing EMLs size. The levels of the investigated factors for the optimized formulation were predicted and the desirability function was computed. The optimized formulation was then prepared for further characterization. Vesicle size and zeta potential of the optimized formula were measured by employing the same method and instrument used for measuring particle size (Section 2.4).

### 2.6. In Vitro FBX Release from the Optimized EMLs Formula

FBX release from the optimized EMLs was investigated as previously described [30]. PBS (pH 7.4) with tween 80 (0.1% *v*/*v*) was utilized to perform the study. A quantity of 2 mg FBX-raw and optimized FBX-EMLs containing 2 mg FBX were introduced into a dialysis bag previously activated (MWCO = 12,000 Da) and then were kept in a shaker water bath at 37 °C. Samples were withdrawn at 0.5, 1, 2, 4, 6, 8, 12, 18, and 24 h time points and then analyzed for FBX content by a previously reported high performance liquid chromatography (HPLC) method [31].

### 2.7. Determination of IC50 by MTT Assay

The IC50 values of HCT 116 colorectal carcinoma cells left untreated (control) or treated with Blank EMLs, pure FBX (FBX-R), or FBX-loaded EMLs (FBX-EMLs) for 24 h, were measured by the MTT [3-(4,5-dimethylthiazol-2-yl)-2,5-diphenyltetrazolium bromide] assay as previously described [32,33]. Briefly, the cell suspension (2 × 10^5^ cells) was seeded into a 96-well tissue culture plate and incubated in a humidified environment (5% CO_2_, 37 °C) to allow the complete cell attachment. At the end of the 24 h treatment, the MTT protocol was applied and the absorbance at 569 nm was read by using a Spark^®^ multimode microplate reader (Tecan Group Ltd., Seestrasse, Maennedorf, Switzerland). The IC50 for Blank EMLs, FBX-R, or FBX-EMLs was calculated based on the curves obtained measuring the variation of cell viability (%) as a function of increasing concentrations (0.39, 1.56, 6.25, 25, 100, and 200 µM) of EMLs, FBX-R, or FBX-EMLs.

### 2.8. Cell Cycle Analysis

The cell cycle analysis was performed by using flow cytometry as previously described [16]. The HCT 116 cells, previously seeded in 6-well plates (3 × 10^5^ cells/well), were left untreated (control) or treated with a sub-µM IC50 (2.1 µM) of Blank EMLs, FBX-R, or FBX-EMLs for 24 h. At the end of the treatment, cells were separated by centrifugation and fixed by using 70% cold ethanol. After an additional centrifugation followed by a washing step with PBS, cells were stained (15 min at room temperature) with a PBS solution containing propidium iodide (PI) (BD Bioscience, San Jose, CA, USA) and RNase staining buffer. At the end of the staining, each sample was analyzed by using a flow cytometer (FACS Calibur, BD Bioscience, San Jose, CA, USA).

### 2.9. Annexin V Staining

Cell apoptosis was investigated by using the reported dual staining technique [16,34]. The HCT 116 cells, previously seeded in 96-well plates (1 × 10^5^ cells/well), were left untreated (control) or treated with 2.1 µM of Blank EMLs, FBX-R, or FBX-EMLs for 24 h. A commercially available Annexin V-FITC Apoptosis Kit (BD Bioscience, San Jose, CA, USA) was used for cell staining following manufacturer’s instructions. Following incubation, the cells were centrifuged and re-suspended in 1X binding buffer (500 µL). Five μL of propidium iodide and Annexin V-FITC, allowing to detect different stages of apoptosis and differentiates apoptosis from necrosis, were added to each well and incubated for 5 min at room temperature in the dark environment followed by flow cytometry analysis.

### 2.10. Caspase-3 Assay

Caspase-3 activity in cells subjected to the different experimental conditions was measured using a commercial kit (BD Biosciences, San Jose, CA, USA). The HCT 116 cells, previously seeded in 96-well plates (5 × 10^4^ cells/well), were left untreated (control) or treated with 2.1 µM of Blank EMLs, FBX-R, or FBX-EMLs for 24 h. At the end of the treatment, the HCT 116 cells were first washed and then lysed by using a cell extraction buffer. Caspase-3 activity was measured in each cell lysate by reading the absorbance at 405 nm by using a Spark^®^ multimode microplate reader (Tecan Group Ltd.).

### 2.11. Mitochondrial Membrane Potential (MMP)

The changes in MMP occurring in HCT 116 cells, previously seeded in 96-well plates (1 × 10^5^ cells/well), left untreated (control) or treated with 2.1 µM of Blank EMLs, FBX-R, or FBX-EMLs for 24 h, were monitored by using a MitoProbe™ TMRM Assay Kit for Flow Cytometry as previously described [33].

### 2.12. Statistical Analysis

The software selected to perform the statistical analysis was IBM SPSS^®^ statistical software (Ver. 25, SPSS Inc., Chicago, IL, USA). In the case of multiple comparisons, one-way or two-way ANOVA along with the Tukey’s *post hoc* test was employed. Each set of experiments, reported as means ± SD, was performed at least four times. Only *p* values < 0.05 were considered statistically significant.

## 3. Results

### 3.1. Experimental Design of FBX-EMLs

#### 3.1.1. Fit Statistics for Sequential Model Selection and Validation

The measured response, particle size (nm, Y), best fitted to the quadratic model. Selection of the sequential model was based on the highest correlation coefficient (*R*^2^) and the lowest predicted residual error sum of squares (PRESS) (Table 3).

In addition, the predicted and adjusted *R*^2^ were in logical accordance, indicating that the selected model was valid. Adequate signal to noise ratio is illustrated by adequate precision value greater than 4, confirming the relevance of the selected model to explore the experimental design space.

**Figure 1:**
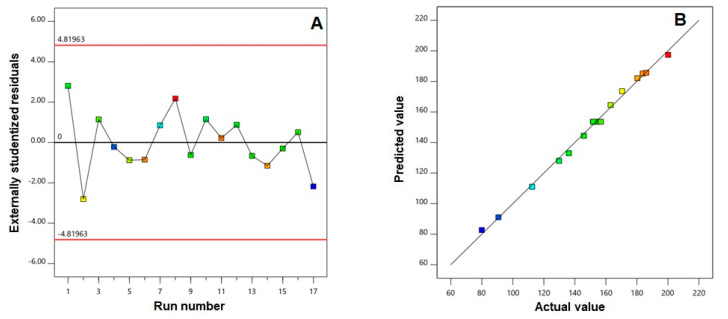
(**A**) Externally studentized residuals vs. run number plot and (**B**) Predicted vs. actual values plot for particle size of FBX-EMLs.

To assess the goodness of fit of the selected model, diagnostic plots were generated for the measured particle size. The residual vs. run plot illustrated in Figure 1A shows that the response is not influenced by any lurking variable as depicted by the randomly dispersed points.

Moreover, the highly linear pattern observed in the predicted vs. actual values of particle size (Figure 1B) confirmed that the observed responses were in good correlation with the predicted ones.

#### 3.1.2. Statistical Analysis for the Effect of Variables on Particle Size (Y)

Nanoparticulate drug delivery systems have turned out as an effective approach for therapeutic moieties delivery for tumor treatment owing to their preferential accumulation in the cancer cells via improved permeation and confinement effect. Accordingly, the physicochemical features of these systems, most importantly size, could have subtle effects on tumor invasion. FBX-EMLs showed nano-sized vesicles with average size ranging from 79.97 ± 0.98 to 200.17 ± 3.98 (Table 2). The EMLs dispersions were uniform and homogeneous as depicted by the relatively small standard deviation. ANOVA for the particle size revealed that the quadratic model was significant as evidenced by its *F*-value of 191.13 (*p* = 0.0024). The lack of fit was not significant (*F* = 3.26; *p* = 0.1420), confirming that the measured size fits to the chosen model. The coded equation representing the quadratic sequential model was generated as follows:

***Y*_1_** = 153.62 + 5.13 X_1_ + 15.16 X_2_ − 42.18 X_3_ − 0.565 X_1_X_2_ + 4.80 X_1_X_3_ + 7.50 X_2_X_3_ − 2.03 X_1_^2^ + 2.29 X_2_^2^ − 8.34 X_3_^2^

**Figure 2:**
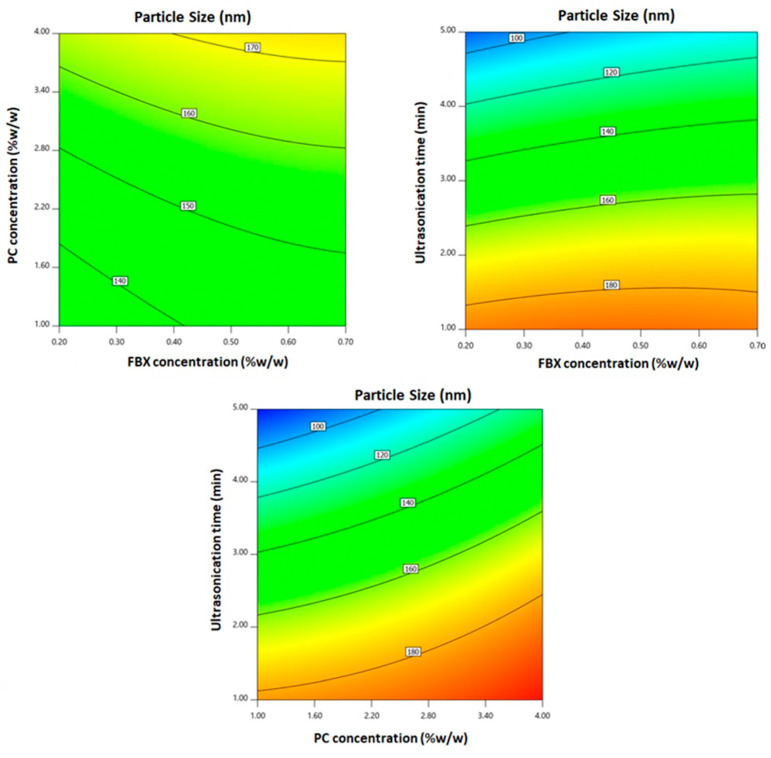
2D-contour plots for the effect of FBX concentration (X_1_), PC concentration (X_2_), and ultrasonication time (X_3_) on the particle size of FBX-EMLs.

According to ANOVA results, all the linear terms (X_1_, X_2_, and X_3_), representing the studied factors, exhibited significant effects on the particle size at *p* < 0.05. In addition, the quadratic term (X_3_^2^), representing the ultrasonication time, and the interaction terms X_1_X_3_ and X_2_X_3_, representing the interaction between ultrasonication time and either FBX or PC concentrations, exhibited significant effects on the particle size at the same level. Two dimensional contour plots for the effects of the investigated variables on particle size are illustrated in Figure 2.

As obvious in Figure 2, the EMLs particle size significantly increases with increasing both FBX and PC concentrations (*p* = 0.0024 and *p* < 0.0001, respectively). This behavior is affirmed by the positive coefficients of both X_1_ and X_2_ in the equation generated in coded terms. The influence of PC concentration was more notable than that of FBX as proved by the higher coefficient of its correlative term. On the other hand, a significant decrease in the particle size was observed with increasing ultrasonication time (*p* < 0.0001).

### 3.2. Optimization of SMV-EMLs

Based on the particle size constraints, numerical optimization was utilized to predict the optimized levels of the variables. Table 4 shows the variables levels and the predicted and observed responses for the optimized formulation.

The optimized formulation fulfilled the criteria of minimized particle size with desirability of 0.997. The % error between the predicted and observed particle size was considerably small, confirming the validity of the optimization technique. Accordingly, the optimized formulation was then subjected for further investigations. The optimized FBX-EMLs showed a zeta potential value of −31.6 mV.

### 3.3. In Vitro FBX Release from the Optimized EMLs Formula

**Figure 3:**
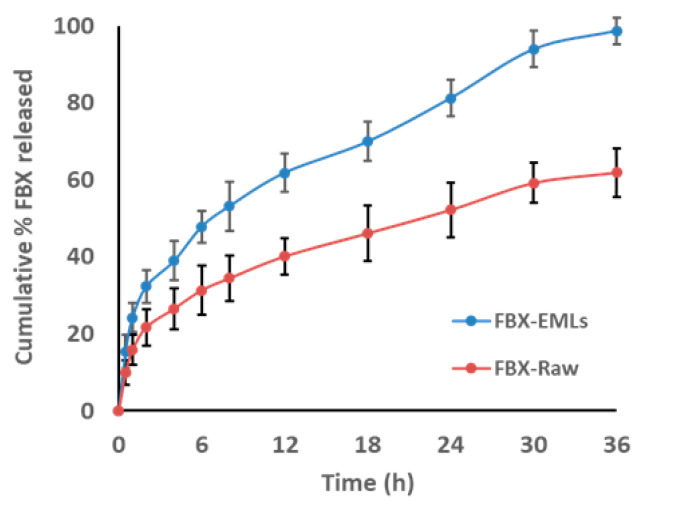
Optimized FBX-EMLs film in vitro release profile in comparison with FBX-R film.

FBX in vitro release resulting from the optimized FBX-EMLs film revealed enhanced release data when compared with FBX-R film (Figure 3).

The optimized FBX-EMLs film released 47.8% ± 3.1 of FBX content within 6 h compared with 31.3% ± 6.3 of FBX release from raw film at the same time point. FBX cumulative % released within 24 h were 81.2% ± 4.7 and 52.2% ± 7.1 for optimized FBX-EMLs and FBX-R films, respectively. The optimized formula released almost all its content, 98.7% ± 3.1, within 36 h, while the FBX-R films reached a percentage equal to 61.95% ± 6.2 after the same period of time.

### 3.4. Optimized FBX-EML Formulation Shows the Lowest IC50 Value

**Figure 4:**
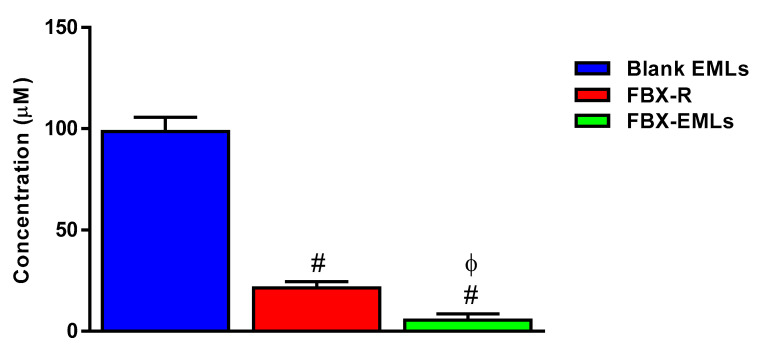
IC50 of the Blank EMLs, FBX-R, and FBX-EMLs in the HCT 116 cells. Data are the mean of 4 independent experiments ± SD. ^#^ Significantly different vs. Blank EMLs (*p* < 0.05); ^Φ^ Significantly different vs. FBX-R (*p* < 0.05).

The MTT assay was carried to investigate the pharmacological activity and the toxic potential, expressed as IC50, of Blank EMLs, FBX-R, and FBX-EMLs treatments (24 h) on HCT 116 cells. As expected, the highest IC50 value thus, the lowest toxic potential was observed in HCT 116 cells treated for 24 h with the empty carrier (Blank EMLs) (IC50 = 98.6 ± 7.1 µM) (Figure 4).

The drug in the absence of the carrier (FBX-R) showed a significantly higher toxic potential (lower IC50 value) compared to Blank EMLs (IC50 = 21.4 ± 3.0 µM; *p* < 0.05). The highest toxic potential was measured in the case of the drug encapsulated into emulsomes (FBX-EMLs); in fact, the IC50 value of this optimized formulation was equal to 5.4 ± 3.1 µM, about 4-fold lower compared to FBX-R (*p* < 0.05). Based on these results, we selected the FBX-EMLs concentration (2.1 µM), a sub-µM IC50, and studied its effects on cell cycle phases, percentage of apoptotic and necrotic cell populations, MMP, and caspase-3 concentration. A comparison with the same concentration of not encapsulated drug (FBX-R) was also carried out.

### 3.5. FBX-EMLs Treatment Inhibits the Proliferation of HCT 116 Cells

**Figure 5:**
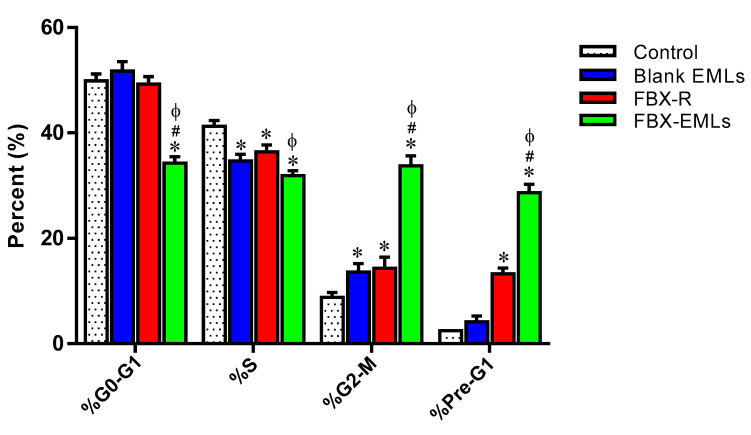
Impact of Blank EMLs, FBX-R, or optimized FBX-EMLs treatments on HCT 116 cell cycle phases. Data are the mean of 4 independent experiments ± SD. * Significantly different vs. Control (*p* < 0.05); ^#^ Significantly different vs. Blank EMLs (*p* < 0.05); ^Φ^ Significantly different vs. FBX-R (*p* < 0.05).

Figure 5 reports the effects of the different experimental treatments (Blank EMLs, FBX-R, or optimized FBX-EMLs) on HCT 116 cell cycle phases.

In the case of untreated (control) cells, % values for G0/G1, S, G2-M, and Pre-G1 phases equal to 49.9 ± 1.3%, 41.3 ± 1.1%, 8.8 ± 0.9%, and 2.5 ± 0.1%, respectively, were measured, clearly indicating quick proliferative properties for the samples incubated in the absence of treatment. There was a slight but significant (*p* < 0.05) modulation only in the case of S phase (34.7 ± 1.3%) and G2-M phase (13.6 ± 1.6%) for the treatment with Blank EMLs compared to control cells. Instead, FBX-R treatment was able to induce significant cell cycle changes compared to control cells (*p* < 0.05), except for G0/G1, with a reduction of the percent of cells in the S phase (36.4 ± 1.3%) paralleled by an increase of the number of HCT 116 cells in G2-M (14.3 ± 2.1%) and Pre-G1 (13.3 ± 1.1%) phases. Of note, the treatment with the optimized FBX-EML formula was able to significantly inhibit the proliferation of HCT 116 cells compared to all the other treatments (*p* < 0.05 vs. all), with significant and very relevant changes (higher number of cells) occurring on G2/M (33.8 ± 1.9%) and pre-G1 (28.7 ± 1.6%) phases, accompanied by a significant reduction of cells in G0/G1 (34.3 ± 1.2%) and S (31.9 ± 0.9%) phases.

### 3.6. The Encapsulation of FBX into EMLs (FBX-EMLs) Strongly Enhances the Pro-Apoptotic Potential of the Drug

**Figure 6:**
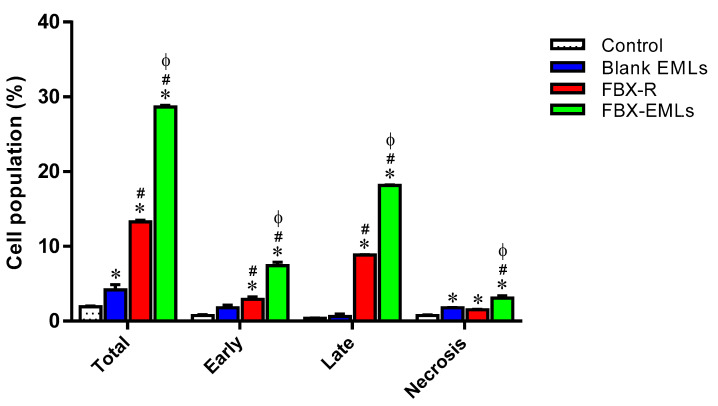
Impact of Blank EMLs, FBX-R, or optimized FBX-EMLs treatments on the percentage of apoptotic or necrotic HCT 116 cells. Total = apoptosis + necrosis; Early = early apoptotic phase; Late = late apoptotic phase. Data are the mean of 4 independent experiments ± SD. * Significantly different vs. Control (*p* < 0.05); ^#^ Significantly different vs. Blank EMLs (*p* < 0.05); ^Φ^ Significantly different vs. FBX-R (*p* < 0.05).

With the aim to better understand whether the potentiated anti-proliferative effect of FBX-EMLs treatment was also combined with pro-apoptotic activities, the impact of the different treatments on the percentage of apoptotic or necrotic HCT 116 cells was examined. Figure 6 depicts the effects of the different treatments on HCT 116 cell status.

The treatment with Blank EMLs did not significantly influence the percentage of cells in both apoptotic stages, while a slight but significant (*p* < 0.05) increase in necrosis (1.8 ± 0.1%) compared to control cells (0.8 ± 0.1%) was observed. FBX-R-treated HCT 116 cells showed a significant enhancement (*p* < 0.05) in apoptosis (early: 2.9 ± 0.3%; late: 8.8 ± 0.1%) compared to both control (early: 0.8 ± 0.1%; late: 0.4 ± 0.1%) and Blank EMLs-treated cells (early: 1.8 ± 0.4%; late: 0.6 ± 0.3%). As expected based on our previous results, the treatment of HCT 116 cells with FBX-EMLs significantly increased the percentage of cell population in early (7.4 ± 0.5%) and late (18.2 ± 0.1%) stages of apoptosis, in necrosis (3.1 ± 0.3%) as well as in apoptosis + necrosis (indicated as total) (28.7 ± 0.2%) compared to all the other experimental conditions (*p* < 0.05), including the treatment with FBX-R, suggesting an enhancement of the pro-apoptotic activity of FBX when encapsulated into the EMLs.

### 3.7. Caspase-3 Activity Increases Following the Treatments with the Free Drug (FBX-R) and the Optimized Formulation (FBX-EMLs)

**Figure 7:**
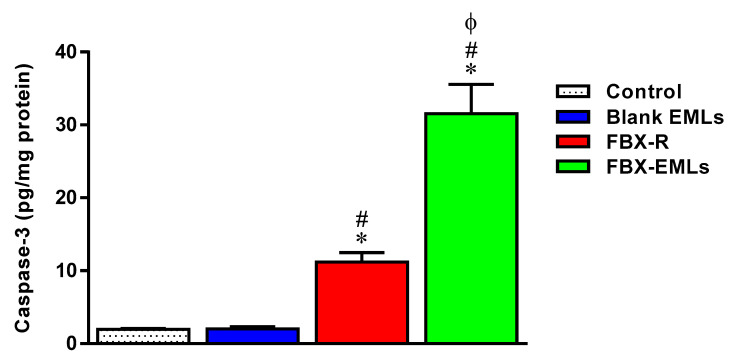
Effect of Blank EMLs, FBX-R, or FBX-EMLs treatments on caspase-3 enzyme contents in HCT 116 cells. Values are expressed as pg/mg of protein. Data are the mean of 4 independent experiments ± SD. * Significantly different vs. Control (*p* < 0.05); ^#^ Significantly different vs. Blank EMLs (*p* < 0.05); ^Φ^ Significantly different vs. FBX-R (*p* < 0.05).

As expected based on the results described in Figure 6, the treatment of HCT 116 cells with the EMLs only (2.0 ± 0.3 pg/mg protein) did not lead to any significant changes in the caspase-3 content compared to control cells (2.0 ± 0.1 pg/mg protein) (Figure 7).

The treatment of HCT 116 cells with FBX-R induced a significant increase of caspase-3 compared to control cells (11.2 ± 1.3 pg/mg protein) (*p* < 0.05). The maximal enhancement in caspase-3 content was observed in the case of the treatment with optimized FBX-EMLs (31.6 ± 4.0 pg/mg protein) (*p* < 0.05 vs. all the other experimental conditions), that was about 3- and 15-folds higher than that of FBX-R-treated and control cells, respectively.

### 3.8. The FBX Ability to Decrease the MMP is Enhanced When Encapsulated into the EMLs

**Figure 8:**
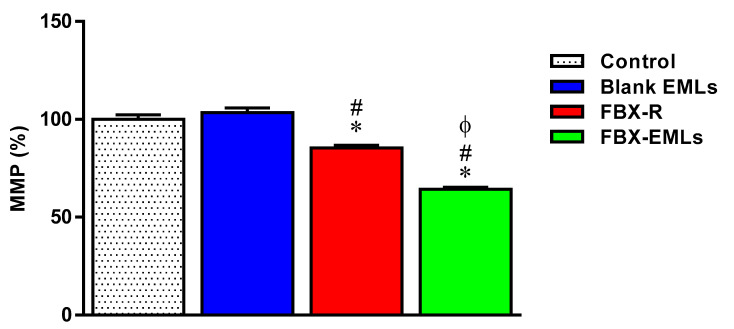
Impact of the treatment with Blank EMLs, FBX-R, or optimized FBX-EMLs on the variation of the MMP (%) of HCT 116 cells. Values were normalized with respect to control untreated HCT 116 cells and are expressed as the percent (%) variation of MMP. Data are the mean of 4 independent experiments ± SD. * Significantly different vs. Control *(p* < 0.05); ^#^ Significantly different vs. Blank EMLs (*p* < 0.05); ^Φ^ Significantly different vs. FBX-R (*p* < 0.05).

No significant changes in MMP (%) were observed between control (100 ± 2.3%) and Blank EMLs-treated (103.4 ± 2.3%) HCT 116 cells (Figure 8).

The treatment with FBX-R significantly decreased the MMP (%) (85.4 ± 1.4%; *p* < 0.05) compared to both Blank EMLs-treated and control cells. The FBX ability to decrease the MMP (%) was significantly enhanced when the drug was encapsulated into the emulsomes (FBX-EMLs) (64.3 ± 1.1%; *p* < 0.05 vs. all the other experimental conditions).

## 4. Discussion

Febuxostat (FBX) is an inhibitor of xanthine oxidase enzyme currently used for the treatment of hyperuricemia in subjects with gout [35]. It has been shown that this drug is practically insoluble in water, while its solubility increases in solvents such as dimethylsulfoxide [36], and the use of drug delivery systems such as EMLs [33,37] could represent a powerful tool to increase its solubility and, at the same time, improve the drug dissolution rate, then enhancing the clinical efficacy of this drug.

The aim of the experimental design and optimization process was to investigate the factors affecting EMLs formulation aiming to obtain a minimum vesicle size (<100 nm). This goal was achieved by predicting the optimized formulation that showed vesicle size of 77.89 nm (Table 4). Particle size increase due to increasing drug concentrations could be ascribed to increased drug loading. While the increased particle size at increased PC concentrations could be explained based on an increased number of formed multiple bilayers. On the other hand, a significant decrease in the particle size was observed with increasing ultrasonication time (*p* < 0.0001) (Figure 2). This reduction could be ascribed to the main principle underlying sonication process. The ultrasound mechanical waves of sonication forms cavitation bubbles in the formulation dispersions. Bubbles of sizes nearly similar to the resonant size for the applied frequency begins to vibrate non-linearly, leading to bubble breakdown; this action provokes extremely high temperatures, high pressures, and shock waves. Thus, the ultrasonic imparted high energy causes size reduction. Based on this explanation, the generated energy increased with increasing sonication times resulting in reduced particle size. This reduction represents a key point to improve drug effects; in fact, different approaches related to pharmaceutical particle technology are often employed with the aim to improve the low aqueous solubility of drugs limiting the in vivo bioavailability after administration [38]. The need to specifically increase FBX poor solubility to derive maximum therapeutic efficacy is also shown in several other works as in the case of Kumar et al. [39] and Kuchekar et al. [40].

The results of zeta potential of the optimized formula showed a value of −31.6 mV that indicates a long-term stability of FBX-EMLs formula. Other factors as the small size of the prepared vesicles and nature of lipid components of the prepared formula contributed to the improved penetration of FBX-EMLs into the cells when compared with FBX-R.

The release profile from optimized FBX-EMLs film showed improved release pattern when compared with FBX-R film (Figure 3). The cumulative amount of FBX released from the optimized film formula could be considered as satisfactory regarding the low solubility of FBX when compared with FBX-R film. Since cancer patients often encounter severe adverse effects due to the poor water solubility of anti-cancer drugs leading to the necessity to administer high doses [41], a drug delivery system allowing for the administration of lower doses of drugs while maintaining effective intracellular concentrations will enhance the therapeutic power of the drug.

Recently, several studies have been conducted to examine the FBX anti-cancer activity [16,42,43], with particular regard to its ability to enhance cancer cells death via apoptosis and decrease the chemotherapy resistance, then representing a promising candidate for cancer treatment. Additionally, based on its ability to reduce uric acid production in the body, this drug has been used for the treatment of tumor lysis syndrome, a metabolic impairment that arises in cancer patients [15,44].

Based on the above evidence, we carried out in vitro cell experiments in which the encapsulation of FBX into EMLs was performed to enhance the toxic potential of the drug towards human colorectal carcinoma (HCT 116) cells, a well-known experimental model in cancer drug discovery to predict clinical efficacy of anti-cancer drugs [26,27,28,29]. As a first step, we determined the cytotoxic potential of the free drug (FBX-R) as well as of the optimized formula (FBX encapsulated into EMLs, FBX-EMLs) on HCT 116 cells, expressed as IC50, the concentration of the drug able to reduce the cell viability by 50%, an index frequently employed to compare the anti-proliferative activity and the toxic potential of different anti-cancer drugs [45]. As showed in Figure 4, the IC50 of FBX-R (21.4 ± 3.0 µM) was significantly decreased by the encapsulation of the drug into the EMLs (5.4 ± 3.1 µM, about 4-fold lower), underlining the enhanced therapeutic potential of the optimized formulation in counteracting cancer cells proliferation. These results are in agreement with recent data showing the ability of EMLs-based formulations to enhance the cytotoxic effects of another “repositioned drug” (simvastatin) on breast cancer cells [33]. Taking into consideration the frequently reported adverse events related to the use of this drug [46], the development of new formulations able to boost its therapeutic effect, reducing the effective dose of FBX could be of great relevance for future translational studies in cancer patients. Additionally, it is also worth noting that, as showed very recently by us, drugs encapsulated into emulsomes have relatively week cytotoxic activity against non-cancerous cells compared to HCT 29 and HCT 116 colon cancer cells (five to six times higher) [47], hence having two possible therapeutic implications represented by the enhanced cytotoxicity towards cancer cells and the reduction of unwanted side effects in healthy non-cancerous cells.

With regard to the ability of the free or EMLs-encapsulated drug to inhibit tumor cells proliferation, the maximal inhibitory activity was observed when treating HCT 116 cells with FBX-EMLs; in fact, the inhibitory effect of FBX-R was significantly strengthened in the presence of the drug delivery system (FBX-EMLs), with significant and very relevant changes (% of cell population) occurring in each of the cell cycle phases, especially in the case of G2/M and pre-G1 phases (Figure 5). The anti-proliferative activity of the optimized formula, underlined by the reduction of the transition from G1 to the S phase and the inhibited transition from G2 to M phase, was paralleled by an enhancement of the pro-necrotic and, to a greater extent, pro-apoptotic potential (Figure 6), as also indicated by the increased caspase-3 content detected after the treatment of HCT 116 cells with FBX-EMLs (31.6 ± 4.0 pg/mg protein) (Figure 7). This anti-proliferative ability of FBX towards cancer cells is in agreement with the results obtained by Oh et al. [48] showing that FBX is able to inhibit breast cancer cell migration and pulmonary metastasis in the hyperlipidemic condition, also strengthening the possible use of xanthine oxidase inhibitors for the treatment of cancer. Most probably the observed enhanced cytotoxic effects observed when employing the optimized formula are coming from the ability of EMLs to enhance the poor solubility of FBX, ensuring a higher intracellular availability [25,49] and a sustained release of the drug [20,49].

As a last step, the ability of the free or EMLs-encapsulated drug to modulate the MMP, representing a well-known index of cell suffering and death, in our human colorectal cancer cell model was tested. Despite the well-demonstrated resistance of cancer cells to MMP-induced changes [33,50,51], both FBX-R and FBX-EMLs treatments were able to significantly decrease the MMP (%) in HCT 116 cells (Figure 8). Of note, the ability of the free drug to decrease the MMP (%) was greatly enhanced (from −14.6 to −35.7%, about 2.4-fold stronger) when FBX was delivered in the presence of EMLs. These data are in agreement with the results showed in Figure 5, Figure 6 and Figure 7 and are of particular relevance since the ability of a molecule/drug to decrease the MMP has been identified as one of the driving forces that lead to: (1) a more robust release of apoptotic factors; (2) a higher tumor cell death rate; (3) a reduced resistance of cancer cells to conventional anti-cancer therapies [52,53].

Overall, our results clearly depict the enhanced cytotoxic activity exerted by the FBX drug when encapsulated into the EMLs on the well-characterized human colorectal carcinoma (HCT 116) cells [26,27,28,29], making the base for the investigation of this optimized formula in vivo.

## 5. Conclusions

In the present study, the optimized formulation of FBX encapsulated into emulsomes (FBX-EMLs) was obtained by Box-Behnken design. This formulation showed minimized vesicle size and enhanced drug release when compared to FBX-R, suggesting the EMLs ability to enhance the dissolution of FBX drug. The in vitro experiments carried out on human colorectal carcinoma (HCT 116) cells clearly demonstrated as the encapsulation of the FBX into the drug delivery system, represented by EMLs, significantly improved all the parameters related to the toxic potential of the drug towards cancer cells, including the IC50 decrease, the enhancement of anti-proliferative activity, the increase of the percentage of apoptotic and necrotic cell populations paralleled by an increment of intracellular caspase−3 concentration, and, finally, the decrease of MMP in cancer cells. Our optimized FBX-EMLs formulation might therefore represent a novel and useful tool for drug repositioning and to improve drug development in colorectal cancer.

## Figures and Tables

**Table 1:** Independent variables and responses used in the Box-Behnken design for the formulation and optimization of febuxostat into emulsomes (FBX-EMLs).

**Independent Variables**	**Levels**		
	(−1)	(0)	(+1)
X_1_: FBX concentration (% *w*/*w*)	0.20	0.45	0.70
X_2_: PC concentration (% *w*/*w*)	1.00	2.50	4.00
X_3_: Ultrasonication time (min)	1.00	3.00	5.00
**Responses**	**Desirability Constraints**		
Y_1_: Particle size (nm)	Minimize		

Abbreviations: FBX, febuxostat; PC, phosphatidyl choline.

**Table 2:** Experimental runs, variables levels, and observed responses of FBX-EMLs prepared according to Box-Behnken design.

Experimental Run #	Independent Variables			Particle Size (nm) ± SD
	FBX Concentration (% *w*/*w*)	PC Concentration (% *w*/*w*)	Ultrasonication Time (min)	
F1	0.20	1.00	3.00	136.15 ± 2.89
F2	0.70	4.00	3.00	170.48 ± 2.63
F3	0.45	2.50	3.00	156.76 ± 1.67
F4	0.20	2.50	5.00	90.70 ± 1.19
F5	0.20	4.00	3.00	163.07 ± 2.34
F6	0.20	2.50	1.00	183.80 ± 3.11
F7	0.70	2.50	5.00	112.42 ± 1.93
F8	0.45	4.00	1.00	200.17 ± 3.98
F9	0.45	2.50	3.00	151.81 ± 2.11
F10	0.45	4.00	5.00	129.80 ± 1.45
F11	0.70	2.50	1.00	186.07 ± 2.47
F12	0.70	1.00	3.00	145.82 ± 2.19
F13	0.45	2.50	3.00	151.68 ± 1.72
F14	0.45	1.00	1.00	180.32 ± 2.27
F15	0.45	2.50	3.00	152.72 ± 1.99
F16	0.45	2.50	3.00	155.13 ± 1.33
F17	0.45	1.00	5.00	79.97 ± 0.98

Abbreviations: FBX, febuxostat; PC, phosphatidyl choline; SD, standard deviation.

**Table 3:** Fit model statistics of FBX-EMLs responses prepared according to Box-Behnken design.

Responses	Model	Sequential *p*-Value	Lack of Fit *p*-Value	*R* ^2^	Adjusted *R*^2^	Predicted *R*^2^	Adequate Precision	PRESS	Significant Terms
*Y: Particle size (nm)*	Quadratic	0.0046	0.1420	0.9959	0.9907	0.9522	47.66	813.53	X_1_, X_2_, X_3_, X_1_X_3_, X_2_X_3_, X_3_^2^

**Table 4:** Optimized variable levels of the optimized FBX-EMLs and its predicted and observed responses.

Variables	X_1_: FBX Concentration (% *w*/*w*)	X_2_: PC Concentration (% *w*/*w*)	X_3_: Ultrasonication Time (min)
Optimum values	0.21	1.07	4.88
Vesicle size (nm)	Predicted value	Observed value	Error %
	77.94	77.89	3.52
